# Molecular Analysis of Stromal Cells-Induced Neural Differentiation of Mouse Embryonic Stem Cells

**DOI:** 10.1371/journal.pone.0166316

**Published:** 2016-11-10

**Authors:** Ramila Joshi, James Carlton Buchanan, Sailaja Paruchuri, Nathan Morris, Hossein Tavana

**Affiliations:** 1 Department of Biomedical Engineering, The University of Akron, Akron, Ohio 44325, United States of America; 2 Department of Chemistry, The University of Akron, Akron, Ohio 44325, United States of America; 3 Department of Epidemiology and Biostatistics, Case Western Reserve University, Cleveland, Ohio 44106, United States of America; University of Hong Kong, HONG KONG

## Abstract

Deriving specific neural cells from embryonic stem cells (ESCs) is a promising approach for cell replacement therapies of neurodegenerative diseases. When co-cultured with certain stromal cells, mouse ESCs (mESCs) differentiate efficiently to neural cells. In this study, a comprehensive gene and protein expression analysis of differentiating mESCs is performed over a two-week culture period to track temporal progression of cells from a pluripotent state to specific terminally-differentiated neural cells such as neurons, astrocytes, and oligodendrocytes. Expression levels of 26 genes consisting of marker genes for pluripotency, neural progenitors, and specific neuronal, astroglial, and oligodendrocytic cells are tracked using real time q-PCR. The time-course gene expression analysis of differentiating mESCs is combined with the hierarchal clustering and functional principal component analysis (FPCA) to elucidate the evolution of specific neural cells from mESCs at a molecular level. These statistical analyses identify three major gene clusters representing distinct phases of transition of stem cells from a pluripotent state to a terminally-differentiated neuronal or glial state. Temporal protein expression studies using immunohistochemistry demonstrate the generation of neural stem/progenitor cells and specific neural lineages and show a close agreement with the gene expression profiles of selected markers. Importantly, parallel gene and protein expression analysis elucidates long-term stability of certain proteins compared to those with a quick turnover. Describing the molecular regulation of neural cells commitment of mESCs due to stromal signaling will help identify major promoters of differentiation into specific cell types for use in cell replacement therapy applications.

## Introduction

The adult central nervous system has a minimal capacity to replace neural cells damaged or lost due to injury or disease.[1] As such, treatment of neurodegenerative diseases has to primarily rely on external interventions including cell replacement therapies.[2] Cell-based therapies of traumatic injuries of the central nervous system or neurodegenerative disorders requires extensive production of specific neural lineage cells. Embryonic stem cells (ESCs) and induced pluripotent stem cells (iPSCs) provide promising cell sources for neural cell therapies due to their capability to generate specific subtypes of neural precursors such as dopaminergic cells, motoneurons, GABAergic cells, astrocytes, and oligodendrocytes.[3] Neural cells derived from ESCs and iPSCs have produced some encouraging results in animal models in terms of tissue integration, functional recovery without teratoma formation, behavioral improvement, and animal survival.[4–6] Efforts to regenerate neural tissue will greatly benefit from experimental approaches to efficiently differentiate stem cells into specific and functional neural cells.

There are several approaches to derive neural progenitors or differentiated neuronal and glial cells *in vitro* by the means of directed differentiation of ESCs. These methods aim to mimic the multistep process of embryonic neural cell development from early stage neural induction, to terminally differentiated neuronal and glial cells. ESCs may be cultured in suspension to form multi-cellular aggregates known as embryoid bodies that differentiate in the presence of retinoic acid.[7] This method is not specific and results in cells from all three germ layers.[8] Additionally, retinoic acid hampers the natural neural patterning and maturation.[9,10] ESCs cultured as a monolayer or in suspension under serum free conditions or in defined media supplemented with growth factors can also yield neural cells but with a relatively low efficiency.[11, 12] The third approach to induce neural differentiation is co-culturing of ESCs with specific bone marrow-derived stromal cells.[13,14] Both intercellular contacts and paracrine signaling from the stromal cells contribute to neural differentiation of ESCs,[15] mimicking embryonic development of the nervous system in terms of direct intercellular contacts and signaling, avoids differentiation-inducing chemicals, and yields specific populations of nerve cells.[16] A limitation of this approach is potential contamination with stromal cells when harvesting differentiated neural cells for transplantation, although this could be avoided using sorting techniques to separate stromal cells from the differentiated cell population. Moreover, mechanisms of stromal cells-mediated neural differentiation are not completely defined yet. Past studies mainly focused on transplantation of co-culture derived neural cells in rodent models, [17,18] and the importance of intercellular contacts between stromal and ES cells on neural differentiation.[19] Molecular drivers of neural cell differentiation and temporal changes in the neural commitment of stem cells in this co-culture environment remain unexplored.

Although neural fate commitment of ESCs *in vivo* is not completely understood, growing efforts to control the ES-stromal cells microenvironment have helped identify transcriptional and epigenetic regulation of neural cell differentiation.[18,20] Several studies have investigated the neurogenesis pathway *in vitro*. In a study comparing neural differentiation of a mouse iPSC line and two mouse ESC lines, no differences were found in the differentiation pathway followed by these cell lines. Moreover, this study identified more than 1000 differentially-expressed factors with roles in neural cell morphology, synaptic transmission, neurogenesis, and neuron recognition.[20] mRNA expression analysis of differentiation of neural crest-like cells from human and primates ESCs *in vitro* helped understand molecular and cellular events in human neural crest development. [19]

Currently, a detailed analysis of molecular events that describe specification of neural progenitors and their differentiation to neurons and glial cells in mES-stromal cells co-culture system is still lacking. Such analysis will broaden our understanding of the neurodevelopmental process, elucidate niche-specific regulators of neurogenesis, and help develop more efficient ESC-based strategies for the treatment of neurodegenerative diseases in future. We address this need by performing a comprehensive molecular analysis of transition of mouse ESCs to specific neural cells in an ES-stromal cells co-culture system by studying temporal changes in the expression of marker genes and proteins specifying pluripotent cells, neural progenitors, and terminally-differentiated neurons, astrocytes, and oligodendrocytes. Understanding molecular regulation of mESCs differentiation in this co-culture system may enable design of strategies to derive defined neural cell types.

## Materials and Methods

### A. Culture of Cells

Mouse embryonic stem cells (mESCs) (EB5, Riken) were maintained on 0.1% gelatin-coated dishes in a medium consisting of Glasgow Minimum Essential Medium (GMEM, Life Technologies) supplemented with 1% fetal bovine serum (FBS, Sigma), 10% knockout serum replacement (KSR, Life Technologies), 2 mM glutaMAX (Life Technologies), 0.1 mM non-essential amino acids (NEAA, Life Technologies), and 1 mM sodium pyruvate (Life Technologies). Final concentrations of 0.1 mM 2-mercaptoethanol (Life Technologies) and 2000 U/ml leukemia inhibitory factor (Millipore) were added to the cell culture medium. A stromal cell line derived from mouse skull bone marrow, PA6 (Riken), was used in co-culture with mESCs to induce differentiation. PA6 cells were maintained on gelatin-coated dishes in αMEM (Life Technologies) supplemented with 10% FBS and 1% antibiotic (Life Technologies). All experiments were conducted with the first 10 passages of both PA6 cells and mESCs.

### B. Preparation of Stromal Cells and Co-Culture with mESCs

PA6 cells seeded at a density of 4×10^4^ cells on gelatin-coated 35 mm Petri dishes grew to a confluent layer within 2 days. The stromal cell layer was mitotically inactivated by treating with 10 μg/ml mitomycin-c (Sigma) for 2 hrs. PA6 cells were then washed three times with PBS and incubated overnight at 37°C and 5% CO_2_ in a “differentiation medium” containing GMEM supplemented with 10% KSR, 2 mM glutaMAX, 0.1 mM NEAA, 1 mM sodium pyruvate, and 0.1 mM 2-mercaptoethanol before co-culturing with mESCs.

mESCs were suspended in the differentiation medium and seeded over the PA6 layer at a density of 1×10^4^ cells per dish. mESCs adhered to the underlying PA6 cells, proliferated over the 2-week culture period to form colonies, and showed neural differentiation. The cell culture medium was renewed on days 4 and 6 and every day thereafter. The cells were cultured for 8 days in the differentiation medium and for an additional week with addition of 1X N2 (Life Technologies).

### C. Quantitative Real Time Polymerase Chain Reaction (q-PCR) Analysis of Marker Genes

Experimental samples were lysed every day for two weeks using a TRK lysis buffer (Omega Biotek) and homogenized by passing through homogenizer mini columns (Omega Biotek). Total RNA was isolated from the samples using an RNA isolation kit (Omega Biotek). DNase was removed using RNase-free DNase kit (Omega BioTek). Purity and concentration of isolated RNA was assessed using OD 260/280 spectrophotometry (Synergy H1M, Biotek instruments). cDNA was synthesized from 1 μg of total RNA using random hexamer primers (Roche).

Real time q-PCR was performed in a Lightcycler 480 II instrument using a SYBR Green Master Mix (Roche).[21] Briefly, 50 ng of cDNA was combined with forward and reverse primers and the SYBR green Master Mix diluted, to a final volume of 15 μl. The reactions were pre-incubated at 95°C for 5 min followed by 45 cycles of amplification, i.e. at 95°C for 10 sec, at 60°C for 10 sec, and at 72°C for 10 sec. Specific primer sequences for all the 26 genes investigated are listed in S1 Table. Expression levels of mRNA for different marker genes were calculated relative to GAPDH using the ΔΔC_t_ method and the fold change in mRNA expression was determined according to the 2^-ΔΔCt^ method. [21,22] q-PCR was also performed on PA6 cells only for several neural marker genes as a negative control.

### D. Immunofluorescence, Imaging and Image Analysis

Co-cultures of mESCs and PA6 cells were fixed every day from day 1 to day 15 with 3.7% formaldehyde for 15 min, followed by three washes with PBS, each for 5 min. The samples were blocked with 5% donkey serum and then incubated overnight at 4°C with primary antibodies. Immunocytochemistry was performed for Nestin with affinity purified chicken Nestin antibody (1:200) (Neuromics), TuJ with rabbit monoclonal class III β-tubulin antibody (1:2000) (Biolegend), glial fibrillary acidic protein (GFAP) with affinity purified chicken IgY (1:2000) (Neuromics), and tyrosine hydroxylase (TH) with rabbit monoclonal TH antibody (Abcam). Secondary antibodies raised in donkey and fluorescently conjugated with Aminomethylcoumarin (AMCA) (1:100), Rhodamine red (1:100), and Alexa Fluor 488 (1:800) (Jackson Immunoresearch) were used to visualize expression of proteins. Ten random areas from each dish were imaged at 20X using an inverted fluorescent microscope (Axio Observer, Zeiss). Consistent magnification and exposure time were used to capture images each day for each marker. Resulting images were used to quantify expression of proteins.

For the protein markers Nestin, TuJ, and GFAP, net fluorescence intensity of the immunostained image was measured after subtracting the background using the “subtract background” tool in ImageJ. Additionally, neural differentiation of mESCs stained with Nestin, TuJ, and GFAP was quantified using an adaptive thresholding technique in ImageJ. Each raw image was converted to a binary image and the total white pixel count, which accounts for the total area of positive staining in an image, was measured. Total number of TH-stained cells in each image was counted using a plugin in ImageJ.

### E. Statistical Analysis

All experiments were performed in triplicates for q-PCR and in duplicates for immunostaining. For protein expression analysis using immunofluorescence, ten random images were captured and analyzed each day. Statistical analyses of protein expression levels were done using one-way ANOVA and Fisher’s post hoc test in Minitab 16 software. Statistical significance was defined at *p* < 0.05.

Two different approaches were used to explore the trajectories of the genes over time. First, an agglomerative hierarchical cluster analysis with the complete linkage method was used to divide the genes into separate clusters.[23] For each gene, daily -ΔΔC_t_ values (i.e., the log_2_ relative expression levels) were first centered by subtracting the mean of -ΔΔC_t_ values based on the two weeks of experiments and daily -ΔΔC_t_ values (i.e., subtracting the mean of all data points from each individual data point). Resulting values were then scaled by the standard deviation of -ΔΔC_t_ values. This allowed the variation of all the genes analyzed over time to be on a similar scale. Random trajectories of -ΔΔC_t_ values were reduced to a set of finite principal components scores by using a second method of functional principal components analysis (FPCA). [24,25] This method helped characterize dominant modes of variations of gene expression profiles based on the overall trajectory of gene expression.[26] Both analyses were performed using the R statistical programming language.

## Results and Discussion

### A. Proliferation and Differentiation of mESCs

**Fig 1:**
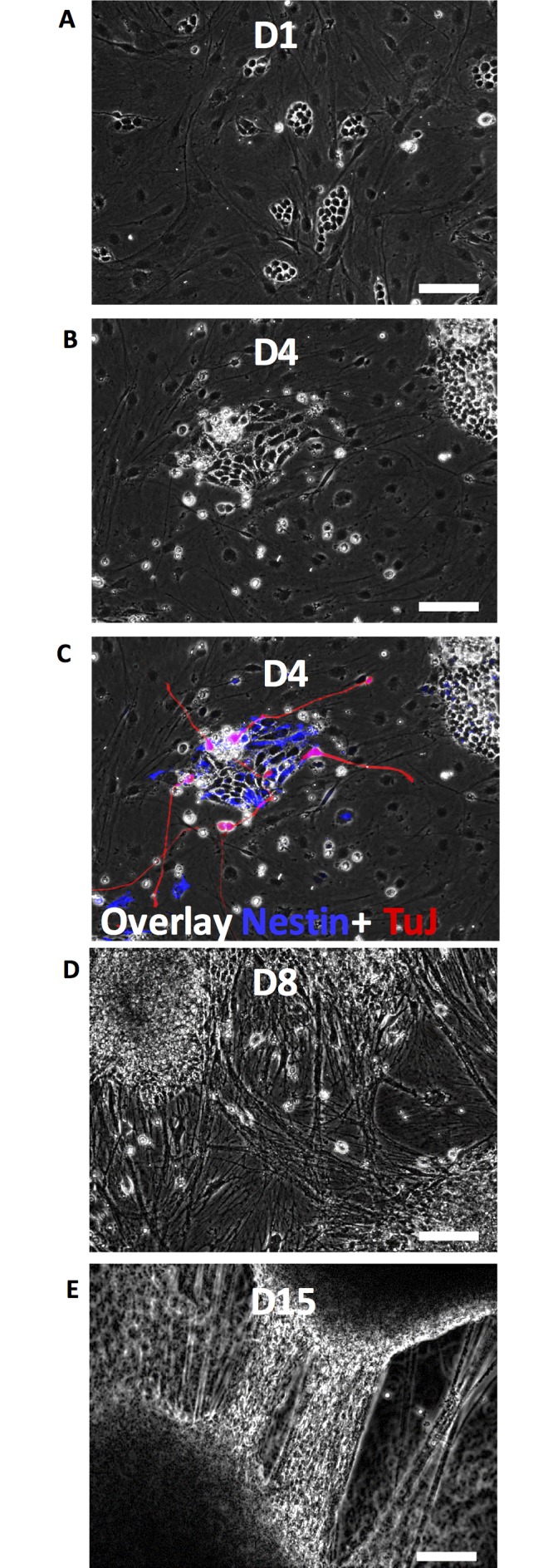
Phase images of differentiating mESCs. (A-E) mESCs in co-culture with stromal PA6 cells proliferate and differentiate to neural cells. Panel (C) shows a composite image produced by overlaying the phase image of panel (B) with immunostaining for neural cell markers Nestin (blue) and TuJ (red). Neurite processes and thick bundles emerge between mESC colonies during the second week of culture. Scale bar: 100 μm. Abbreviations: mESCs, mouse embryonic stem cells; Tuj: class III β tubulin.

mESCs adhered to the PA6 cell layer within a few hours (Fig 1A). Cells showed rapid proliferation and formed distinct colonies (Fig 1B). Direct contact between mESCs and stromal cells was sufficient to induce neural cell differentiation. Within the first 4 days of co-culture, colonies contained differentiating cells that stained positive for neural cell markers (Fig 1B and 1C). With rapid cell division and migration of differentiating cells towards the periphery of each colony, the interspaces between colonies were gradually occupied by differentiating cells (Fig 1D). Neurite processes extended out from differentiating cells, morphologically resembling neural progenitor cells. By the end of the two-week culture period, extensive processes stretched between neighboring mESC colonies and formed thick neurite bundles (Fig 1E).

### B. Gene Expression Analysis of Stromal Cells-Induced Neural Differentiation of mESCs

We conducted a time course gene expression profiling of differentiating mESCs in co-culture with stromal PA6 cells by performing daily q-PCR for two weeks. A set of 26 gene markers of pluripotency, neural progenitors, specific neural cells, and mesodermal markers were used to quantify their mRNA levels. Threshold cycle for each transcript was determined using the second derivative maximum analysis in LightCycler 480 software. Threshold cycle for each gene was normalized to the threshold cycle of the reference gene GAPDH to calculate ΔC_t_.[27] Each value of ΔC_t_ was then normalized with respect to ΔC_t_ of day 0, i.e., undifferentiated mESCs, to calculate ΔΔC_t_. Fold change values were then generated as 2^-ΔΔCt^. Controls that did not contain cDNA template and controls that contained mRNA prior to reverse transcription did not result in amplified sequences. S1 Fig shows sample relative fold change data for several genes. This set of information was generated for all 26 genes from samples collected daily over the two weeks of culture. We note that q-PCR performed on samples from PA6 cells only for several marker genes did not give any positive fold change (S2 Fig). We used hierarchical cluster analysis and functional principal component analysis (FPCA) approaches to track time trajectory of expression of these genes in differentiating mESCs. These analyses were performed on pluripotency and neural cell markers with the exclusion of CNP (an oligodendrocyte marker) that displayed very little relative fold change, i.e., less than 2 folds.

### C. Hierarchical Clustering of Gene Expression Data

**Fig 2:**
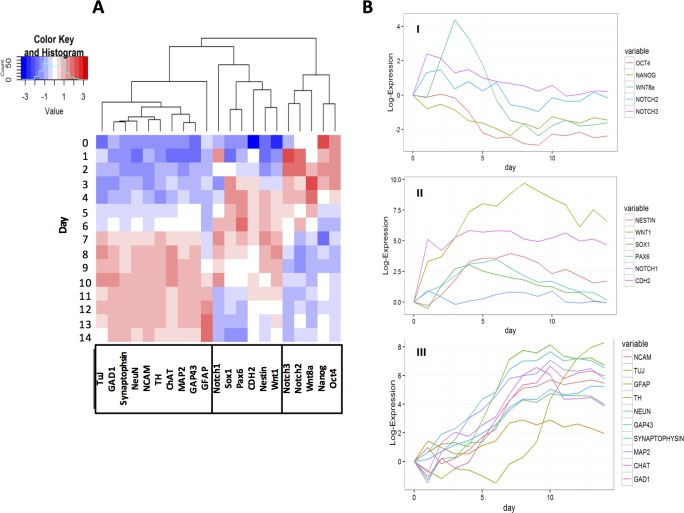
Gene expression trajectory over two weeks of differentiation. (A) Heatmap of the standardized expression trajectories for genes over a two-week co-culture of mESCs andPA6 cells. The genes fall into 3 different clusters that represent genes associated with pluripotency, neural progenitor cells, and specific neural cells. (B) Temporal log2-expression (non-standardized) levels for genes from each of the three clusters.

To elucidate maturational state of developing neural population, we examined gene expression of cells over the course of differentiation process using q-PCR and biostatistical analyses. Fig 2A shows a heatmap of log-scale standardized expression of genes over culture period. Applying an agglomerative hierarchical cluster analysis, we segregated the marker genes into three distinct groups. These 3 clusters reflect distinct cellular stages in the process of obtaining terminally-differentiated neuronal and glial populations from mESCs.

Genes in the smallest cluster showed high expression during the first five days followed by a rapid decrease. This cluster was comprised of Oct4, Nanog, Wnt8a, Notch2, and Notch3 (Fig 2A and Fig 2B-I). Genes in this group are pluripotency markers and quickly down-regulate at the onset of differentiation. Oct4 and Nanog are transcription factors that regulate self-renewal of pluripotent stem cells. Down-regulation of Oct4 and Nanog causes rapid loss of self-renewal capacity of ESCs and differentiation into multiple cell lineages. Moreover, a model by Jaenisch and Young proposes that Oct4, Nanog, and Sox2 co-operatively regulate the network for self-renewal and pluripotency of ESCs.[28,29] Wnt8a, a protein from Wnt family, promotes growth and proliferation of ESCs and prevents their migration; therefore it is expected to be enriched in undifferentiated population of ESCs.[30] High expressions of these markers during the first few days followed by a steady decline in our experiments indicates that mESCs initially proliferate by symmetric divisions and then gradually undergo differentiation via asymmetric divisions when kept in culture with PA6 cells.[31] Expansion of mESCs during the 2-week culture period and migration of cells toward the periphery of colonies during culture with PA6 cells corroborate with the expression pattern of these genes.

Notch2 and Notch3 belong to the Notch signaling network that regulates interactions between physically adjacent cells and pluripotency of stem cells by inhibiting differentiation until a correct cue becomes available.[32] Additionally, Notch proteins restrain the expression of proneural genes and promote differentiation into glial subtypes to regulate the ratio of neurons and glial cells.[33] We did not observe a significant increase or fluctuations of these two genes in our experiments. Considering that Notch2 and Notch3 promote gliogenesis over neurogenesis, their low expression levels in our experimental systems suggest that the stromal cells-induced differentiation favors neurogenesis. Therefore, these Notch proteins were mainly involved in cell patterning and maintaining cells at a proliferative state.

The second cluster consists of genes that highly express between days 4 and 11 and then either downregulate or level off. Temporal expression patterns of Wnt1, Nestin, Cdh2, Pax6, Sox1, and Notch1 representing this group (Fig 2B-II) show that expression of the former three genes remains fairly high for days 12 to 15 while the remaining markers steadily decline. Genes in this cluster that represent the onset of neural differentiation have specific functions in differentiation of ESCs and early patterning of the nervous system.[34,35] Pomp et al. found that the expression of neural crest markers in human and primates ESCs peaked during the first week of culture and gradually decreased during successive weeks. This implies that PA6 cells induce neural crest-like cells before neuronal differentiation and the expression of several neural crest marker genes is downregulated upon differentiation of neural crest derivatives.[36]

Pax6 and Sox1 are transcription factors active in neural stem/progenitor cells and regulate transition of neural stem cells to specific neuronal and glial progenitors, respectively.[37–39] Therefore, their activity is expected to rise in differentiating ESCs, remain high in neural progenitors, and downregulate as cells develop into specific neural cells. The mESCs-PA6 system perfectly reproduced this pattern (Fig 2A and Fig 2B-II). In agreement with the above description, maximum Pax6 activity is delayed with respect to maximum Sox1 levels. Notch1 is known to induce glial differentiation and obstruct neuronal differentiation of ESCs.[40] Therefore, it is expected to be present in neural stem cells but not extensively in neuronal precursor cells. Rise of Notch1 activity during differentiation of mESCs (Fig 2A) agrees with this description; however, low levels of Notch1 (Fig 2B-II) suggest that this co-culture system does not favor glial differentiation of mESCs. Significantly greater expression of Pax6 that favors neuronal differentiation over production of glial cells supports this conclusion.

Wnt1 belongs to the canonical Wnt family that promotes proliferation of cells during central nervous system development and helps induce sensory and midbrain dopaminergic neurons.[41] *In vitro*, Wnt signaling induces neuronal and astroglial differentiation of ESCs but suppresses oligodendroglial differentiation.[42] Therefore, early rise of Wnt1 expression and presence of high levels of this marker throughout culture suggests that Wnt1 supports neural cell commitment of mESCs in this co-culture system and promotes dopaminergic neuron and astrocyte differentiation, consistent with immunostaining results shown below. Another marker in this cluster is Cdh2 or N-cadherin, which is an intercellular junctional protein that maintains beta-catenin signaling during cortical development, regulates Wnt signaling, and induces radial glial progenitor cells.[43] Rise in N-cadherin expression up to day 6 followed by a slight decline suggests a potential role for N-cadherin in inducing neuroectodermal cells from mESCs. This is consistent with a report by Haque et al. that showed an increase in N-cadherin expression in mES and miPS cells between days 4–10.[44] Nestin is a filament protein of neural stem cells responsible for self-renewal and axonal growth of neural stem cells.[45] This is consistent with our experimental observations of radial extension of neurites from differentiating cells in colonies (Fig 1D and 1E). Several studies have shown a rise in Nestin expression during the first week of culture after onset of neural differentiation and a gradual decline thereafter.[46,47] The gradual decrease in Nestin expression in our co-culture system and increase in specific neuronal and glial markers (the third cluster) implies an event similar to neuro-gliogenesis where Nestin is replaced by neurofilaments and GFAP.[48]

The third and the largest cluster represents a group of genes that includes TuJ, GAD1, Synaptophysin, NeuN, NCAM, TH, ChAT, MAP2, and GFAP. The expression of these genes significantly increases as the culture progresses and then levels off, with the exception of GFAP that continuously elevates throughout culture. NCAM encodes for a membrane-bound glycoprotein on neural cells and promotes cell-cell adhesion and neurite outgrowth and remains expressed in functional neural cells.[49] The rapid rise in NCAM expression that is retained for the entire culture period (Fig 2A and 2B-III) suggests that the mESC-PA6 system highly promotes neural differentiation and maturation. A similar expression profile is observed for TuJ and MAP2 genes, which give rise to microtubular proteins in neural progenitor and post-mitotic neuronal cells to enable extension and stability of neurite processes in growing neural cells.[50,51] This is consistent with production of neurites by differentiating cells in colonies (Fig 1D and 1E), and increase in their density and generation and maturation of neural cells shown with immunostaining results below.

In addition to these neuronal and glial progenitor markers, this cluster contains a number of specific markers of functional/terminally differentiated neural cells such as dopaminergic neuronal marker, TH, GABAergic/glutamatergic neuronal marker, GAD1, synaptic marker, Synaptophysin, cholinergic neuronal marker, ChAT, astrocyte marker, GFAP, and neuronal markers, NeuN and GAP43. These specific neural cell markers showed their highest expression levels during the second week of culture. TH is an enzyme that induces formation of L-DOPA in dopaminergic neurons.[52] During differentiation, TH expression starts approximately around day 6 and gradually decreases after day 11. The TH expression pattern in this co-culture model suggests that the stromal cell-mediated neural differentiation recapitulates dopaminergic neural development *in vivo* where TH expression peaks at the end of early embryonic stage and drops off in the late embryonic stages.[53] Interestingly, the expression of astrocyte marker GFAP elevated several days after other genes in this cluster. This is consistent with developmental process of central nervous system where gliogenesis follows neurogenesis to promote axonal guidance and synaptic support.[54] Our findings on TH and GFAP expression were identical to previous observations made by Kitajima et al. who reported onset of TH^+^ dopaminergic and GABAergic marker expressions on day 7. Moreover, gliogenesis, marked by GFAP expression, succeeded neurogenesis in their set up too.[46] Increased expression of Synaptophysin, a synaptic vesicle glycoprotein supporting synaptic transmission, suggests that neuronal cells resulting from this culture system are functionally active, capable of generating synapses, and potentially capable of transmitting signals.[55] Synaptophysin expression elevates within three days of culture and peaks during the second week, correlating with extensive outgrowth of interconnected neurites. High expression levels of GAP43, which encodes for a growth-associated cytoplasmic protein present in growth cones of developing neurons,[56] corroborate with the generation of specific neuronal cells indicated by the expression of markers such as ChAT and GAD1. This highlights the potential of this technique to generate multiple types of terminally differentiated neuronal cells.

To ensure the specificity of stromal cell-derived neural differentiation of mESCs, we performed q-PCR analysis on several markers of mesodermal cells (S3 Fig). We observed only minimal fold change in the expression of mesodermal genes NKX 2.5, GATA4, FLK1 and PECAM. This indicates that unlike the EB-based approach, the mESC-PA6 system is highly specific and primarily guides mESCs differentiation to neural cells. Our results are consistent with gene expression profiling of differentiating embryonic stem cells during neural development using bayesian networks in terms of identifying and differentiating temporal changes of markers of pluripotency and stage-specific neural cells.[57] A similar approach of studying temporal kinetics of cell surface markers and transcription factors expression during neutrophil differentiation of human iPSCs showed stage specific expression profiles that recapitulated embryonic hematopoiesis and revealed unexpected molecular events that could help enhance *in vitro* protocols for iPSC hematopoiesis. [58]

### D. Functional Principal Components Analysis (FPCA) of Gene Expression

**Fig 3:**
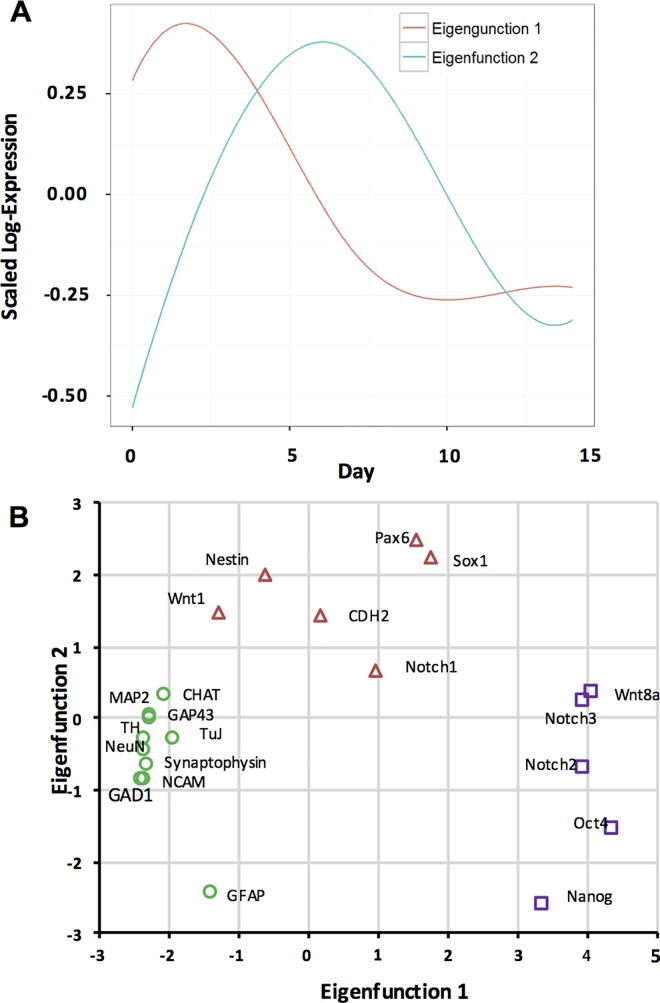
Functional principal component analysis of gene expression. (A) Eigenfunctions (EF) of gene expression are shown over time. The trajectory of each gene may be approximated by an additive combination of EF1 and EF2, and the mean trajectory for all genes. (B) EF1 and EF2 scores for expression of each gene help approximate trajectory of each gene from the two eigenfunctions.

We performed an independent statistical analysis, FPCA, to validate gene expression trajectories resulting from the hierarchical clustering. FPCA and cluster analysis fundamentally differ in that cluster analysis collects genes with a similar expression profile into discrete clusters, whereas FPCA emphasizes variations in expression of genes to elucidate presence of strong patterns in data. Fig 3A displays the eigenfunctions (EFs) of the genes for the first two FPCs over the two weeks of culture. The expression profile of each gene may be approximated by an additive combination of these two EFs, and the mean trajectory across all genes. Genes with a high EF1 score (Fig 3B) express highly in early days of culture similar to EF1 in Fig 3A. Similarly, genes that score high for EF2 have minimal expression in early days, elevate with increase in culture time and drops again toward the end of second week. For example, the pluripotency genes Oct4 and Nanog that have very high EF1 and low EF2 scores show high expression levels during the first few days and a rapid decline with time. Expression profiles of a group of genes such as Pax6 and Sox1 with high EF2 scores follow a trend similar to the EF2, i.e., low expression in early days followed by a rapid rise and decline toward final days of culture. A cluster of genes including MAP2, ChAT, and GAP43 with a negative EF1 score and an EF2 score of about zero shows a trajectory opposite to the EF1 profile, i.e., very low expression during early days and a rapid rise thereafter, and independent from EF2. Unlike all other markers, GFAP shows negative EF1 and EF2 scores indicating that its expression profile results from adding up EF1 and EF2 graphs and flipping it around the horizontal axis. In other words, the expression of the GFAP gene amplifies towards the end of the culture period (opposite of EF1) and does not elevate in the middle of the culture period (opposite of EF2). This is consistent with the q-PCR data that showed a rapid rise in GFAP expression in a late stage of culture (Fig 2B-III). This analysis based on two principal components segregates the genes into three distinct groups of pluripotency markers, early stage neural cells markers, and late stage terminally differentiated cell markers, and validates the results of our hierarchical clustering.

### E. Protein Expression Analysis of Differentiating mESCs

**Fig 4:**
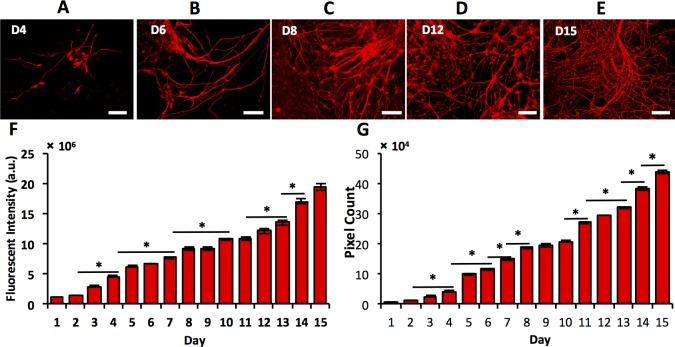
Immunocytochemical analyses of TuJ stained images. (A-E) Differentiating mESCs immunostained for TuJ (beta-III tubulin) on different days of co-culture with PA6 cells. (F) TuJ expression quantified using daily fluorescent intensity measurements show continuous increase in protein content in culture. (G) An independent adaptive thresholding method applied to TuJ-stained images shows increasing TuJ expression over the culture period. Scale bar: 100 μm. * indicates *p*<0.05.

**Fig 5:**
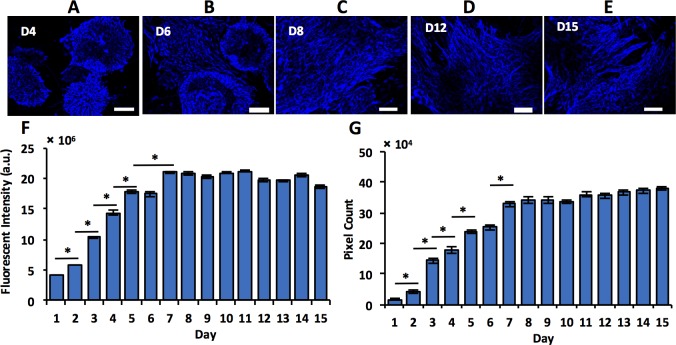
Immunocytochemical analyses of Nestin stained images. (A-E) Differentiating mESCs immunostained for Nestin on different days of co-culture with PA6 cells. (F) Daily fluorescent intensity measurements of Nestin-stained samples show that protein expression increases during the first week of culture but remains steady thereafter. (G) Quantification of Nestin expression using an adaptive thresholding technique. Scale bar: 100 μm. * indicates *p*<0.05.

Next we performed immunostaining of select markers to determine temporal changes in protein expression of differentiating mESCs and elucidate how protein and gene expressions of specific markers may correlate. We conducted daily protein expression analysis of neural stem/progenitor cell markers Nestin and TuJ for the entire culture period, and specific neuronal marker TH and glial marker GFAP during the second week of culture using quantification of both immunofluorescence images and image processing techniques. We note that this method is semi-quantitative as presence of multiple layers of differentiating cells may affect the measured fluorescent intensity. Starting point and duration of this analysis was selected based on gene expression profiles of these markers (S1 Fig). Image analysis based on daily fluorescence intensity measurements of TuJ-stained samples (Fig 4A–4E) showed a continuous increase in the protein content during culture (Fig 4F). In addition, we used an adaptive thresholding method to analyze images of TuJ expression for total neurites density, which takes into account both length and thickness of neurites (similar to Fig 1D and 1E). Neurites density of differentiating cells significantly increased (Fig 4G), with a trend similar to that of fluorescence intensity data. Considering that TuJ is expressed in newly developed post-mitotic neurons to support their maturation and axonal development, continuous increase in TuJ content implies that this co-culture system supports generation of maturing neuronal cells from mESCs. Expression of Nestin in differentiating cells significantly increased up to day 8 of culture but remained steady thereafter (Fig 5A–5G).

These observations are consistent with gene expression patterns of TuJ and Nestin. At a gene level, TuJ expression increased steadily, peaked on day 8, and subsequently leveled off. Continuous increase in the TuJ protein content indicates high translation efficiency of TuJ gene and protein stability in differentiating cells, resulting in an increase in the protein concentration during culture. Nestin gene expression also elevated for the first week of culture but then steadily decreased. However, at a protein level it remained unchanged despite decreasing gene expression, most likely due to the stability of this protein in neural cells.

**Fig 6:**
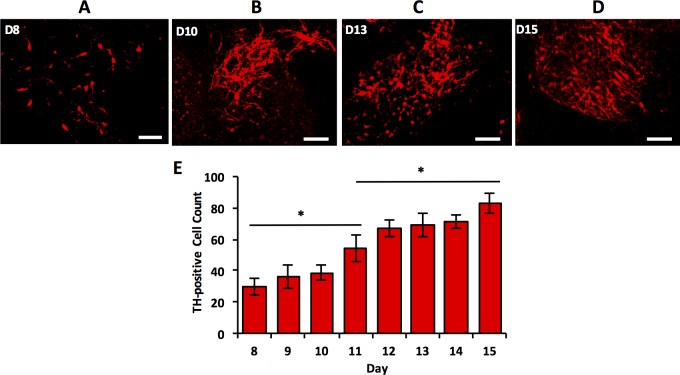
Immunocytochemical analyses of TH stained images. (A-D) Immunostained images of TH-positive dopaminergic neurons during the second week of co-culture of mESCs and PA6 cells. (E) The number of dopaminergic neurons increases with culture time. Scale bar: 100 μm. * indicates *p*<0.05.

**Fig 7:**
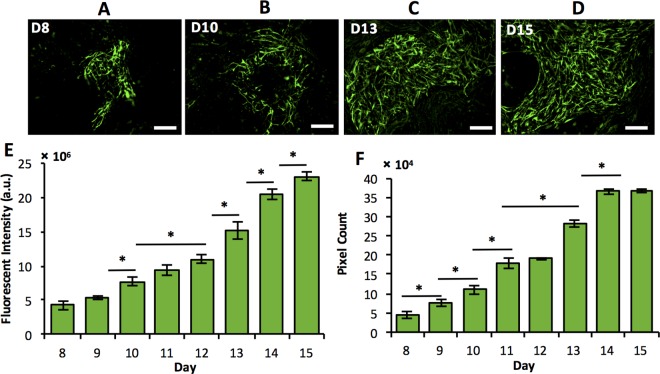
Immunocytochemical analyses of GFAP stained images. (A-D) GFAP-positive astrocyte cells derived from co-culturing of mESCs and PA6 cells. (E) GFAP expression quantified using fluorescent intensity measurements shows a continuous increase in the protein content. (F) Adaptive thresholding of GFAP-stained samples. Scale bar: 100 μm. * indicates *p*<0.05.

Tracking and quantifying daily expression of TH and GFAP proteins in mESC colonies showed differentiation into two main neural sub-lineages of central nervous system. Cells terminally differentiated into dopaminergic neurons and astrocytes as demonstrated by increasing levels of TH (Fig 6A–6D) and GFAP (Fig 7A–7D), respectively. On each day, TH expression was quantified by counting the number of cells stained positive in ten randomly selected locations from the culture. The number of TH^+^ cells consistently increased from day 8 to day 15 (Fig 6E). Again, this agrees well with TH gene expression that elevated during the second half of culture period with a maximum fold change on day 10. However, rise in the protein expression was not as dramatic. This is potentially due to the regulation of translation and protein degradation in TH-expressing cells. Generally, protein abundance reflects its biological roles. Regulatory and secreted proteins such as TH may be produced and degraded rapidly, whereas structural proteins such as TuJ are more stable and present for longer time periods.[59,60] Expression of the astrocyte marker GFAP was quantified by fluorescence intensity and adaptive thresholding approaches (Fig 7E and 7F) that showed a significant rise in protein levels during the second week of culture, consistent with the GFAP gene expression pattern.

This temporal protein expression analysis of differentiating stem cells induced by stromal PA6 cells demonstrated the evolution of neural stem/progenitor cells and specific neural cells, and a close association with their corresponding gene expression profiles. Additionally, simultaneous tracking and comparison of gene and protein expression for individual markers enabled delineating information about long-term stability of certain proteins such as TuJ and Nestin versus those with a quick turnover such as TH. Expanding on this protein expression analysis will elucidate molecular regulation of differentiation of stem cells and generation of specific neural cells due to signaling with stromal cells, and identification of major molecular promoters of differentiation into specific cells.[61]

To the best of our knowledge, there has not been any comprehensive gene and protein expression analysis of neural differentiation of a stromal-ES cells co-culture system before. Several approaches for temporal gene expression profiling during differentiation of stem cells to neural lineages have established stage specific neural lineage markers expression. [61–63] A microarray based whole genome gene expression profiling study showed that a cluster of genes involved in neurites guidance, cerebral and hippocampal neurogenesis, and synaptic transmission were upregulated in differentiated embryoid bodies on day 50. [63] Comparing global gene expression profiles of neural cells derived from mouse ESCs and iPSCs using neurobasal medium supplemented with retinoic acid showed that both cells follow a similar path of differentiation and expression of marker genes throughout the process of acquiring definitive neural cells fate over a culture period of 20 days. Moreover, hierarchical clustering of global gene expression grouped the neuronal populations from both mESCs and miPSCs together.[20] The reported expression profiles in this study closely matches to our observations.

## Conclusions

We tracked dynamic changes at gene and protein levels in mESCs undergoing neural differentiation due to signaling from stromal cells. Immunostaining and real time q-PCR gene profiling coupled with hierarchical clustering and principal component analysis techniques allowed us to capture temporal variations of 26 genes and 4 proteins in this co-culture system. Both hierarchical clustering and FPCA identified three distinct cohorts of genes that describe the transition of cells from pluripotency to specific neural cells. A clear understanding of changes in neuro- and glio-genesis marker genes and proteins during differentiation of ESCs or iPSCs, which have fewer associated ethical questions, will help design new strategies to derive specific cell types for the treatment of neurodegenerative disorders. Additionally, this approach can be used as a quality control measure to assess robustness of neural cell differentiation protocols.[64] This study and future investigations to identify neural differentiation inducing-factors produced by stromal and stem cells in this co-culture system will enable developing novel protocols to generate neural cells as in this co-culture system but without using feeder cells and eliminate concerns regarding contamination of differentiated cells with stromal cells. Further, stem cell-derived neuronal and glial progenitors can serve as a unique model to study genetic modifications,[65] identify and target specific pathways,[66] and screen compounds for large scale drug discovery.[67]

## Supporting Information

S1 Fig Fold changes of select genes are shows over a two-week co-culture of mESCs and PA6 cells. (TIFF)

S2 Fig Fold changes of expression of select genes in PA6 cells is compared to gene expression in pluripotent mESCs (day 0) to ensure that reported fold changes in this study are indeed due to neural differentiation of mESCs. (TIFF)

S3 Fig Fold changes of select mesodermal genes in co-cultures of mESCs and PA6 cells. (TIFF)

S1 Table List and sequence of primers for 26 genes analyzed in co-cultures of mESCs and PA6 cells. (TIFF)
