# Supplementary figures and images for: Molecular Analysis of Stromal Cells-Induced Neural Differentiation of Mouse Embryonic Stem Cells
Source: PLoS One. 2016 Nov 10;11(11):e0166316. doi: 10.1371/journal.pone.0166316 (PMC5104328; doi:10.1371/journal.pone.0166316)

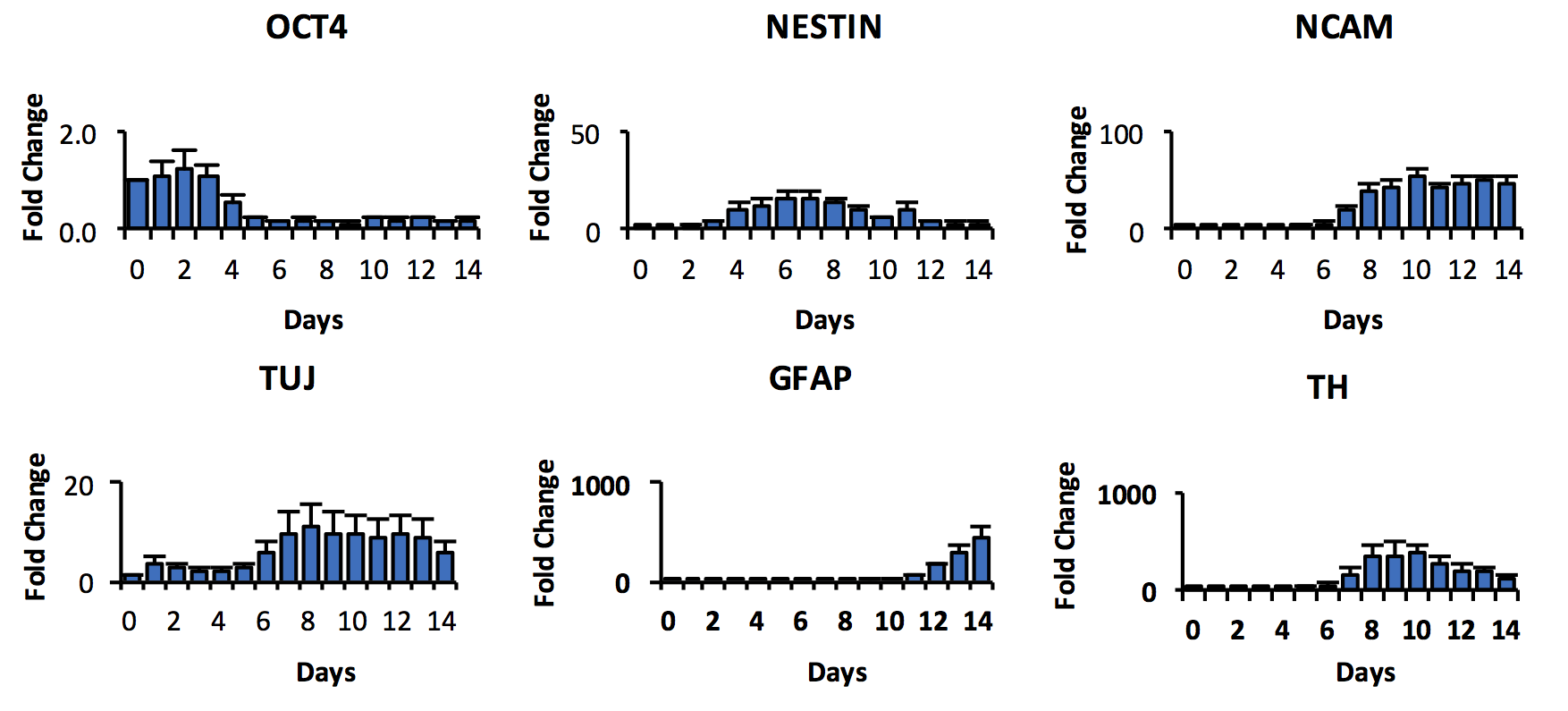

Supplement: S1 Fig — (TIFF) [file pone.0166316.s001.tiff]

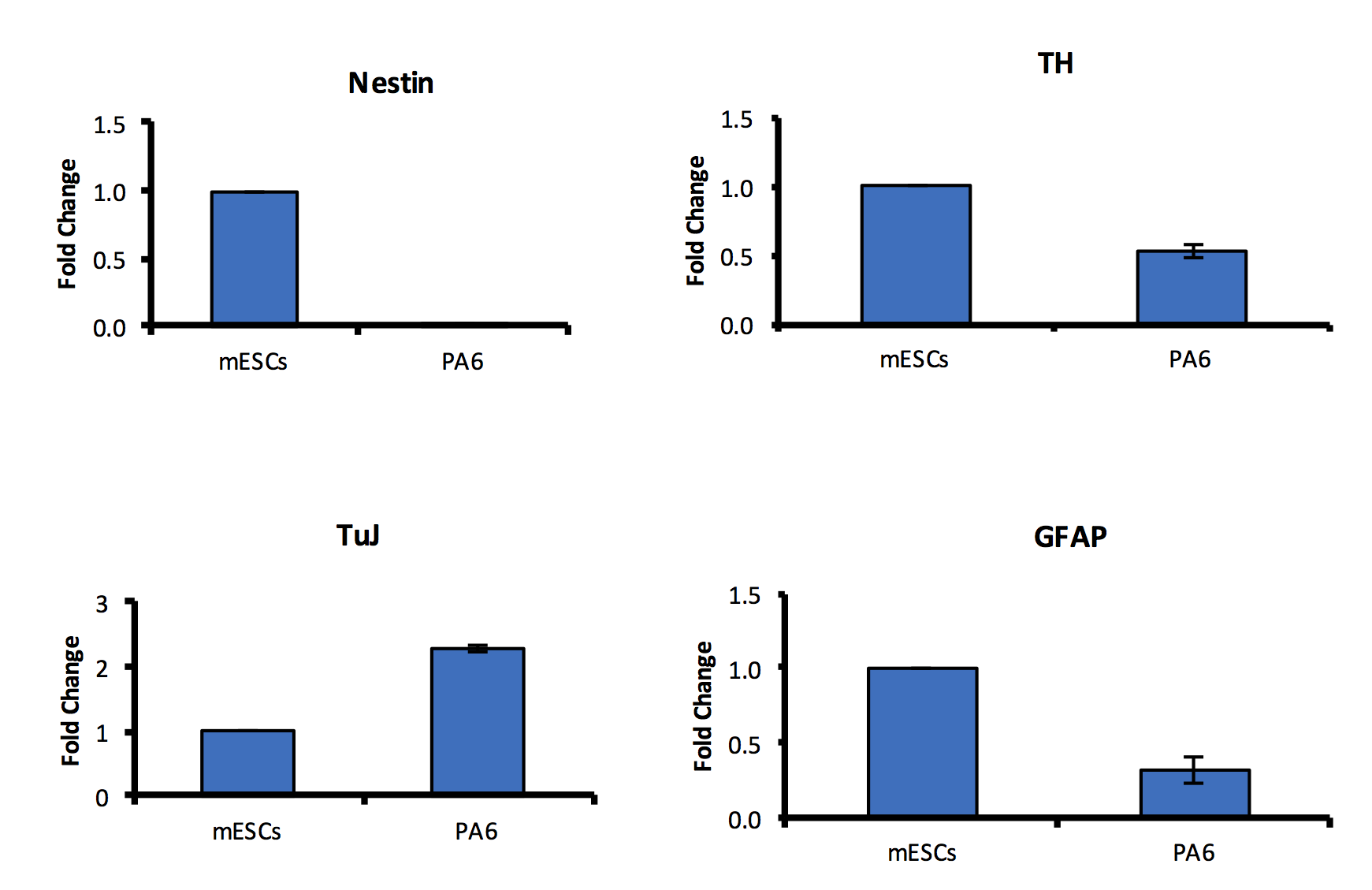

Supplement: S2 Fig — (TIFF) [file pone.0166316.s002.tiff]

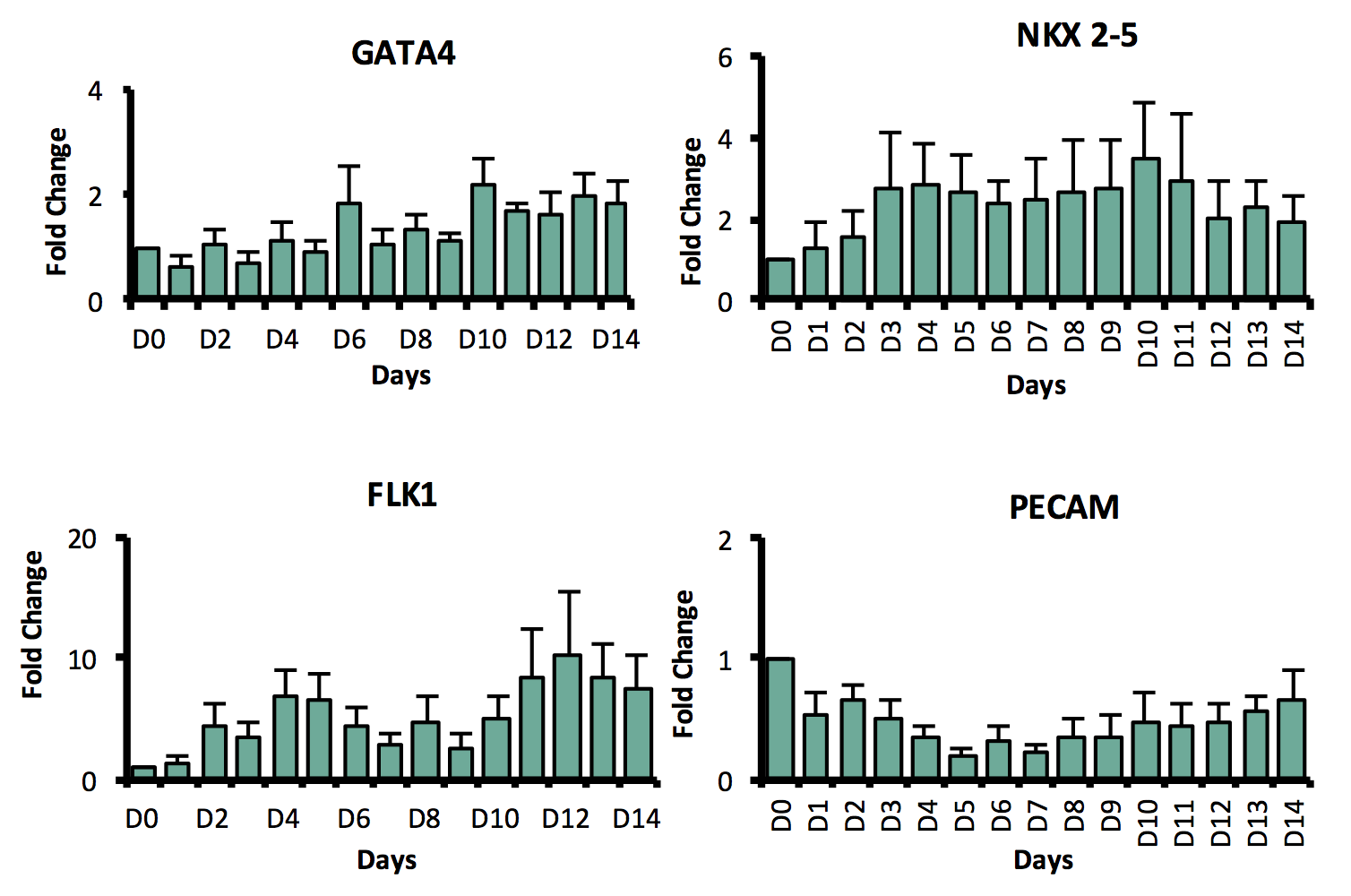

Supplement: S3 Fig — (TIFF) [file pone.0166316.s003.tiff]

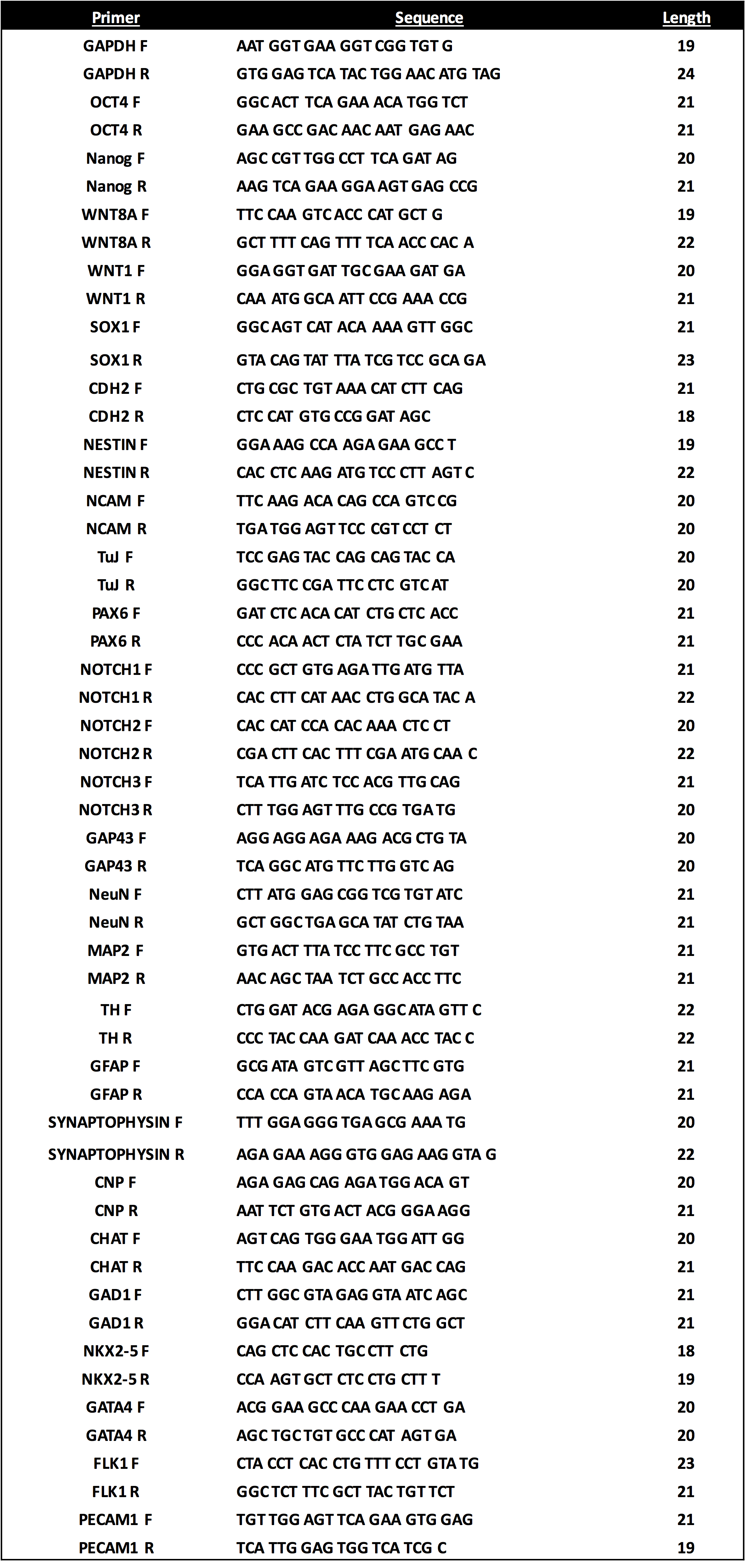

Supplement: S1 Table — (TIFF) [file pone.0166316.s004.tiff]
